# Screening efficacy of PhA and MNA-SF in different stages of sarcopenia in the older adults in community

**DOI:** 10.1186/s12877-022-03716-x

**Published:** 2023-01-09

**Authors:** Xiaoli Zhu, Xinying Dong, Li Wang, Xueting Lao, Shugang Li, Hao Wu

**Affiliations:** 1 Department of Child, Adolescent Health and Maternal Care, School of Public Health, Capital Medical University, No.10 YOU’anmenwai Xitoutiao, Fengtai District, Beijing, 100069 China; 2 Fangzhuang Community Health Center, Capital Medical University, Beijing, 100078 China; 3 School of general practice and continuing education, Capital Medical University, No.10 YOU’anmenwai Xitoutiao, Fengtai District, Beijing, 100069 China

**Keywords:** Community, The older adults, Sarcopenia, The phase angle, MNA-SF

## Abstract

**Objective:**

To compare the screening ability of the phase Angle (PhA) and the Short-Form Mini Nutritional Assessment (MNA-SF) alone and combined detection in the different stages of sarcopenia among the older adults in the community.

**Methods:**

The older adults aged 65 and above were enlisted during community outpatient service and their nutritional status was evaluated by MNA-SF scale. PhA was measured by bioelectrical impedance analysis (BIA). AWGS2019 and EWGSOP2010 were used to define the different stages of sarcopenia. We measured skeletal mass index (SMI) and grip strength with BIA and electronic grip apparatus and measured body function with 6-m pace, SPPB test, and standing test.

**Results:**

The AUC of PhA in the screening of possible sarcopenia was 0.640, the sensitivity was 58.49%, the specificity was 66.67%, and the cut-off value was 4.5. The AUC of the combined PhA and MNA-SF for possible sarcopenia was 0.642, the sensitivity was 57.55%, and the specificity was 70.00%. The AUC of MNA-SF for the screening of pre-sarcopenia was 0.805, the sensitivity was 66.67%, the specificity was 85.83%, and the cut-off value was 12. The AUC of the combined PhA and MNA-SF was 0.826, the sensitivity was 75.00%, and the specificity was 85.00%. The AUC of PhA in the screening of sarcopenia (common type) was 0.808, the sensitivity was 82.35%, the specificity was 73.33%, the cut-off value was 4.4. The AUC of the combined PhA and MNA-SF for sarcopenia (common type) was 0.835, the sensitivity was 76.47% and the specificity was 81.67%. The AUC of PhA and for the screening of severe sarcopenia was 0.935, the sensitivity was 93.33%, the specificity was 92.50%, and the cut-off value was 4.1. The AUC of the combined PhA and MNA-SF was 0.943, the sensitivity was 86.67%, and the specificity was 93.33%.

**Conclusion:**

The screening ability of PhA alone or in combination was higher than that of MNA-SF in the screening of possible sarcopenia. The screening ability of the combined detection was higher than that of PhA alone in the screening of pre-sarcopenia. The combination of PhA and MNA-SF or PhA alone all performed better value in the screening of sarcopenia (common type). Compared to MNA-SF, the PhA performed better in the screening of severe sarcopenia, which provided references for identifying patients with different stages of sarcopenia in the community.

Sarcopenia is a disease which leads to the gradual decrease in skeletal muscle mass by 0.1 to 0.5% annually from the age of 30, and a drastic decrease after the age of 65 [1]. It is estimated that a decrease by about 8% every 10 years until the age of 70, and then decrease by 15% every 10 years [2]. The phase angle (PhA) and the Mini Nutritional Assessment Short Form (MNA-SF) scores mainly reflect the nutritional status of the older adults. When the PhA and the level of MNA-SF is low, the nutritional status of the body is also low. Insufficient protein and energy intake can also lead to the decrease of muscle mass, gradually developing into sarcopenia. The prevalence of sarcopenia in the older adults is 8.4% [3]. The incidence of sarcopenia among the older adults in the community aged 60 and above is 18.0% [4]. The prevalence of sarcopenia in 80-year-older in the United States is 7% in males and 11% in females [5]. Fractures and disabilities caused by sarcopenia seriously influence the quality of life of the older adults in their later years, and bring heavy burdens on individuals, families, and society. Studies have shown that inadequate intake of energy and nutrients is an important cause of sarcopenia. 13-week nutritional supplement intervention helps increase muscle mass and improves lower limb function in senile sarcopenia patients [6]. (PhA) [7] and (MNA-SF) [8] can be used to identify the malnourished older adults early in community, therefore, these two are widely applied in the screening of nutritional status. The PhA and MNA-SF applied in identifying the patients with sarcopenia in early stage have great public health significance in sarcopenia prevention. The AWGS2019 diagnostic consensus proposed the concept of “possible sarcopenia”, presenting as decrease muscle strength with or without physical function, and is specifically used in primary care or community health promotion to achieve early lifestyle interventions [9].

Phase angle (PhA) is a measurement value obtained from bioelectrical impedance analysis (BIA), and high phase angle is a sign of cell membrane integrity [10] and better cellular function [11]. At present, phase angle is applied in studies at home and abroad to assess the nutritional status of patients with diseases [12]. The study reported that the decreased phase angle has certain clinical significance for predicting sarcopenia [13]. Related studies abroad [14] and at home [15] analyzed the diagnostic value of phase angle for sarcopenia in the older adults. Therefore, the phase angle plays an important role in the diagnosis of sarcopenia. As a simple and inexpensive method for evaluating the nutritional status of the older adults, MNA-SF has been widely used in the screening of malnutrition [16] and its prediction in the older adults [17] and it is also adopted to evaluate the nutritional status of patients with sarcopenia [18]. It has been reported that the MNA-SF score decreases gradually with the severity of sarcopenia [19]. Some studies have used MNA-SF test to assess the risk of malnutrition in the hospitalized patients and found that it has a good predictive effect on low muscle-mass index [20].

Although studies have found the role of the phase angle (PhA) and MNA-SF in the diagnosis of sarcopenia, the sarcopenia often goes through the stages of possible sarcopenia, the pre-sarcopenia, the sarcopenia (common type), and severe sarcopenia. Patients in different stages of sarcopenia have different symptoms, and the prevention and treatment measures also vary. Therefore, intervention can be taken after identifying the stage of sarcopenia in the early period, which is of great public health significance in controlling and preventing sarcopenia. So, screening patients with sarcopenia at different stages among the older adults in the community is of great significance. There is no study comparing the efficacy of the MNA-SF and PhA in identifying patients at different stages of sarcopenia, and the efficacy of the combination of MNA-SF and PhA has not been reported yet. Therefore, this study attempted to identify patients at different stages of sarcopenia in the outpatient department of community hospital in accordance with AWGS2019 and EWGSOP 2010 [21]. In addition, receiver operation characteristic (ROC) curve is adopted to figure out the screening ability of PhA and MNA-SF in different stages of sarcopenia, so as to provide reference for the early intervention of sarcopenia.

## Methods

### Study design

This is a cross-sectional study.

### Participants

#### The selection of participants

A total of 270 older adults who visited the outpatient department of Fangzhuang Community Health Service Center in Beijing from October 2021 to December 2021 were enlisted. This study passed the ethical review of Capital Medical University Ethics Committee (Z2021SY014), and all subjects signed informed consent forms.

#### The inclusion and exclusion criteria

The inclusion criteria are as follows: ①Age 65 ~ 85 years old; ②Should be able to communicate③Cooperate with data collection and sign informed consent. The exclusion criteria are as follows: ①Older adults with cognitive (language, sensory perception, etc.) dysfunction or at the onset of mental illness ②Older adults with cardiac pacemaker implantation or joint replacement ③Older adults severe heart, liver, kidney and other important organ dysfunction.

#### Diagnostic criteria for different stages of sarcopenia

Sarcopenia, severe sarcopenia and possible sarcopenia were diagnosed in accordance with the diagnostic criteria published by the Asian Sarcopenia Working Group (AWGS) in 2019. Pre-sarcopenia was diagnosed by the 2010 European Working Group on Sarcopenia in Older People (EWGSOP), thus, as having only lowered muscle mass, reported as Skeletal muscle mass (SMI). ①The cut-off values for having lowered skeletal mass index (SMI) for a measurement with BIA are: male< 7.0 kg/m^2^, female< 5.7 kg/m^2^②Physical function assessment: 6 m gait speed < 1.0 m/s or SPPB score ≤ 9 or standing test ≥12 s③Grip strength: male< 28 kg, female< 18 kg If①and ② or ③are satisfied, sarcopenia (common type) can be diagnosed. If ①and② and ③are satisfied, severe sarcopenia can be diagnosed. Possible sarcopenia is diagnosed as having a decreased hand grip strength or having decreased 6-m gait speed time (Table 1). The control group was defined as older adults without severe sarcopenia, sarcopenia, possible sarcopenia, or pre-sarcopenia.

**Table 1:** The classification of sub-types of sarcopenia

Different stages	SMI ①male< 7.0 kg/m^2^ female< 5.7 kg/m^2^ ②male≥7.0 kg/m^2^ female≥5.7 kg/m^2^	Grip strength ③male< 28 kg, female< 18 kg ④male≥28 kg, female≥18 kg	Physical function assessment⑤6 m gait speed < 1.0 m/s or SPPB score ≤ 9 or standing test ≥12 s ⑥6 m gait speed > 1.0 m/s or SPPB score > 9 or standing test < 12 s
Possible sarcopenia	②AND③OR/AND⑤		
Pre-sarcopenia	①AND④AND⑥		
sarcopenia (common type)	①AND③OR⑤		
severe sarcopenia	①AND③AND⑤		

### Questionnaire survey

A questionnaire was used to collect the basic information of all participants. The nutritional status of the subjects was assessed with the MNA-SF scale, and the functional health status was assessed by the Short Physical Performance Battery (SPPB). All the questionnaires were completed face-to-face to the subjects.

### Height and weight measurements

The height and weight were measured using a stadiometer (BLT-1B0a). During the measurement, the subjects were asked to wear light clothing, removed their shoes and hats, and keep their torso straight. The instrument displays height and weight automatically.

### Grip strength test [22]

Electronic grip force measurement (model WCS-100) produced by Shanggai Xinman Technology Education Equipment Co, LTD was applied. WCS-100 Electronic Grip Strength Tester, also known as Dynamograph, is a new generation of physical measuring instruments. Equipped with pressure difference sensor and micro-computer technology, WCS-100 Electronic Grip Strength Tester (or Dynamograph) provides stable and reliable data display clearly. It can be powered by AC or laminated battery cell of 9 V. The specific method used is as follows, the subjects were asked to stand with elbow straighten at an angle of 90 degrees. They were then asked to bend the elbow and grasp the handle hard. The new measurement peak was displayed in the screen after that. If the subject was unable to stand, the test could also be performed in a sitting position. Grip strength was measured twice by the dominant hand, and the average value was used to evaluate the grip strength [23].

### 6 m gait speed test

A straight-line distance of 9 m was selected on the open and flat ground with marks on the point of 0, 4 and 6 m. The subjects moved forward at normal walking speed after hearing the order from the tester. The tester recorded the time when a foot touches the 0 m mark and passed the 6 m mark respectively.

### Body composition detection (Inbody770)

A comprehensive analysis of the human body can be performed using the Inbody770 from Bass Medical Equipment Trading company (Shanghai). The specific measurement methods are as follows: Subjects were required to fast, refrain from water, and avoid strenuous exercise for 2 h before the test. During the test, subjects should keep quiet, wear light clothes and avoid carrying metal products. Then the subjects were asked to take off their shoes and hats, keeping their torso naturally straight with hands hanging down at a 15-degree angle to the torso. Holding the electrode handle with both hands and standing on the feet electrode of the base. They were asked to press the thumb on the electrode gently following the voice prompts.

### SPPB test [24]

Methods used were hierarchical assessment of standing balance, 4-m walking speed at usual pace, and standing up for five times from a seated position in a chair. The timed results of each sub-test were rescaled according to predefined cut-points for obtaining a score ranging from 0 (worst performance) to 4 (best performance). The total score of SPPB ranges from 0 to 12. For standing balance, participants were asked to remain standing with their feet as close together as possible, then in a semi-tandem position, and finally in a tandem position. Each position had to be held for 10 s. We considered the presence of balance deficit as the inability to maintain tandem position for at least 10 s.

### Statistical analysis

All the survey data were recorded by Epidata3.1, and the database was confirmed after verifying the data. SPSS 25.0 was used for statistical analysis of the survey data. Continuous variables were summarized as means ± standard deviation or median and interquartile range (IQR), and categorical variables were summarized as counts and percentages. Characteristics of participants and their PhA and MNA-SF score were compared using the chi square test, Fisher’s exact test, and Kruskal-Wallis test, where appropriate. Spearman was used to analyze the correlation between variables, with the results as follows: < 0.15 very weak, 0.15–0.25 weak, 0.25–0.4 moderate, 0.4–0.75 strong, > 0.75 very strong [25]. In multivariate analysis, binary Logistic regression model was adopted to analyze the relationship between variables, and the “Enter” method was adopted to introduce independent variables. The ROC curve was applied to assess the screening ability of the PhA and MNA-SF for sarcopenia. Screening ability of the PhA and MNA-SF in identifying different stages of sarcopenia were analyzed. In the combined detection, the screening results of the PhA and MNA-SF were used as independent variables, and the clinically confirmed diagnosis was taken as dependent variables to conduct Logistic regression. The predicted probability of Logistic regression was used to draw the ROC curve. Receiver operating characteristic curve ROC was drawn by MedCalc20.0. The sensitivity and specificity were combined to find the best cut-off value, and the ROC area between groups was compared by Z test. Test level α = 0.05.

## Results

### Participant characteristics

A total of 270 subjects were included in this study, including 120 patients without severe sarcopenia, sarcopenia (common type), possible sarcopenia, or pre-sarcopenia, 106 patients with possible sarcopenia, 12 patients with pre-sarcopenia, 17 patients with sarcopenia (common type), 15 patients with severe sarcopenia, 92 males and 178 females (Table 2).

**Table 2:** Characteristics of study population

Events	Control group (***n*** = 120)	Possible sarcopenia (***n*** = 106)	Pre-sarcopenia (***n*** = 12)	Sarcopenia (common type) (***n*** = 17)	Severe sarcopenia (***n*** = 15)	***P***
**Gender**						0.026
Male	51(42.5)	28(26.4)	6(50.0)	5(29.4)	2(13.3)	
Female	69(57.5)	78(73.6)	6(50.0)	12(70.6)	13(86.7)	
**Family average monthly income**						0.895
≤ 3000	6(5.0)	3(2.8)	0(0.0)	0(0.0)	0(0.0)	
3001–6000	69(57.5)	60(56.6)	8(66.7)	12(70.6)	9(60.0)	
6001–9000	26(21.7)	30(28.3)	1(8.3)	3(17.6)	3(20.0)	
>9000	19(15.8)	13(12.3)	3(25.0)	2(11.8)	3(20.0)	
**Education level**						0.322
Junior high and primary school	29(24.2)	32(30.2)	7(58.3)	5(29.4)	2(13.3)	
High/secondary specialized school	31(25.8)	30(28.3)	2(16.7)	1(5.9)	5(33.3)	
Junior college	36(30.0)	26(24.5)	2(16.7)	5(29.4)	5(33.3)	
Bachelor degree or above	24(20.0)	18(17.0)	1(8.3)	6(35.3)	3(20.0)	
**Pre-retirement occupational**						0.314
Public official	43(35.8)	24(22.6)	5(41.7)	4(23.5)	2(13.3)	
technician	42(35.0)	44(41.5)	3(25.0)	7(41.2)	9(60.0)	
else	35(29.2)	38(35.8)	4(33.3)	6(35.3)	4(26.7)	
**Marital status**						0.006
Married	106(88.3)	86(81.1)	10(83.3)	14(82.4)	7(46.7)	
else	14(11.7)	20(18.9)	2(16.7)	3(17.6)	8(53.3)	
**Living situation**						0.091
Alone	10(8.3)	12(11.3)	1(8.3)	1(5.9)	5(33.3)	
Together	110(91.7)	94(88.7)	11(91.7)	16(94.1)	10(66.7)	
**smoking**						0.159
Yes	11(9.2)	6(5.7)	3(25.0)	1(5.9)	0(0.0)	
No	109(90.8)	100(94.3)	9(75.0)	16(94.1)	15(100.0)	
**drinking**						0.110
Yes	24(20.0)	12(11.3)	3(25.0)	2(11.8)	0(0.0)	
No	96(80.0)	94(88.7)	9(75.0)	15(88.2)	15(100.0)	
**Hypertension**						0.003
Yes	73(60.8)	69(65.1)	7(58.3)	3(17.6)	6(40.0)	
No	47(39.2)	37(34.9)	5(41.7)	14(82.4)	9(60.0)	
**Diabetes**						0.618
Yes	36(30.0)	32(30.2)	2(16.7)	3(17.6)	6(40.0)	
No	84(70.0)	74(69.8)	10(83.3)	14(82.4)	9(60.0)	
**Dyslipidemia**						0.479
Yes	68(56.7)	70(66.0)	6(50.0)	12(70.6)	10(66.7)	
No	52(43.3)	36(34.0)	6(50.0)	5(29.4)	5(33.3)	
Hormone intake						0.162
Yes	4(3.3)	2(1.9)	2(16.7)	0(0.0)	0(0.0)	
No	116(96.7)	104(98.1)	10(83.3)	17(100.0)	15(100.0)	
Protein intake						0.217
Yes	7(5.8)	7(6.6)	0(0.0)	2(11.8)	3(20.0)	
No	113(94.2)	99(93.4)	12(100.0)	15(88.2)	12(80.0)	
Vitamin D intake						0.815
Yes	19(15.8)	19(17.9)	2(16.7)	2(11.8)	4(26.7)	
No	101(84.2)	87(82.1)	10(83.3)	15(88.2)	11(73.3)	
**age**	70(67,72)	72.5(68.0,77.0)	67.5(67.0,70.0)	73.0(70.5,77.5)	81.0(77.0,82.0)	< 0.001
**height**	164.0(158.0,170.0)	160.0(157.0,165.5)	160.0(157.3168.0)	158.0(153.0,161.0)	156.0(149.0,160.0)	< 0.001
**weight**	67.3(60.0,72.9)	68.3(59.8,76.7)	53.5(50.7,57.3)	53.4(49.7,60.9)	50.1(47.7,54.5)	< 0.001

### Comparison of PhA and MNA-SF in different sarcopenia stages

The PhA and MNA-SF scores were different in different sarcopenia stages(*P* < 0.001). With the severity of sarcopenia, the PhA and MNA-SF score was lower (Table **3**).

**Table 3:** PhA and MNA-SF comparison of different sarcopenia stages

Variables	Control group	Possible sarcopenia	Pre-sarcopenia	Sarcopenia (common type)	Severe sarcopenia
**PhA**	4.797 ± 0.549	4.525 ± 0.558	4.600 ± 0.354	4.200 ± 0.471	3.680 ± 0.549
***F***	17.547				
***P***	< 0.001				
**MNA-SF**	14(13,14)	14(13,14)	12(11,13)	13(12,13.5)	13(12,13)
***Z***	38.297				
***P***	< 0.001				

### Correlation analysis of PhA and MNA-SF with sarcopenia components

The MNA-SF score was moderately positively correlated with SMI (*r* = 0.399, *P* < 0.05). PhA was strongly positively correlated with SMI and grip strength, and moderately positive correlated with walking speed and SPPB score (*r* = 0.539, 0.507, 0.289, 0.333, *P* < 0.01) (Table **4**).

**Table 4:** Spearman correlation analysis between PhA, MNA-SF with sarcopenia components

Events	MNA-SF	PhA	SMI	Handgrip
PhA	0.185**			
SMI	0.399^**^	0.539^**^		
Handgrip	0.106	0.507^**^	0.626^**^	
6 m gait speed	−0.047	0.289^**^	0.063	0.282^**^
SPPB score	−0.020	0.333^**^	0.088	0.302^**^

**P < 0.01，*SPPB* Short physical performance battery

### The screening ability analysis of PhA and MNA-SF in screening of possible sarcopenia

**Fig. 1:**
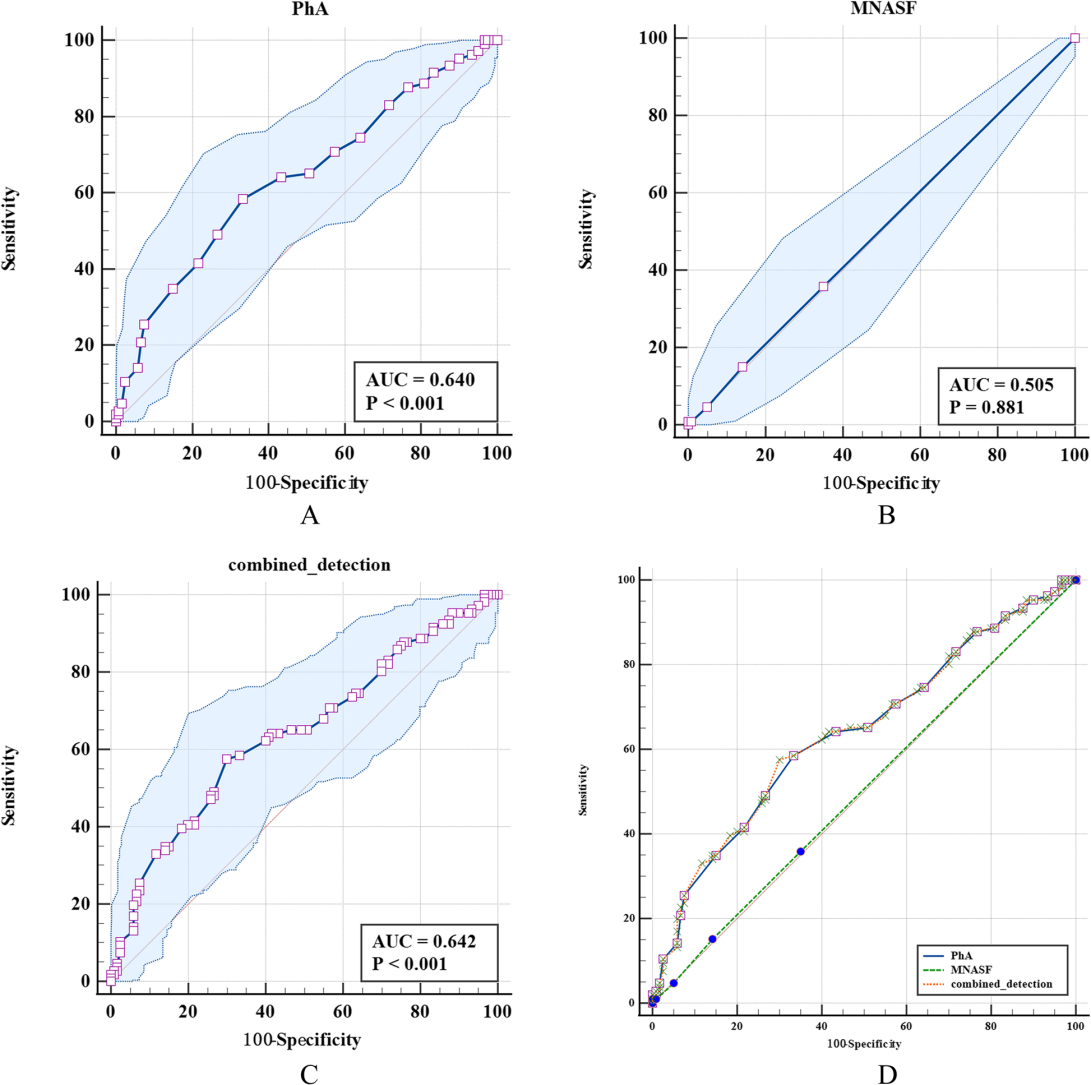
The area under the ROC curve of PhA and MNA-SF single or combined detection for the screening of possible sarcopenia. Note: **A** is the ROC curve for the screening of possible sarcopenia by PhA, **B** is the ROC curve for the screening of possible sarcopenia by MNA-SF, **C** is the ROC curve for combined detection for screening of possible sarcopenia, **D** is the ROC curve for the screening of possible sarcopenia by PhA, MNA-SF and the combined detection

The area under the ROC curve of PhA was 0.640, which was higher than that of MNA-SF at 0.505 (*Z* = 2.804, *P* = 0.005). No difference was found between the combined detection value with PhA alone. The ROC area of combined detection of PhA and MNA-SF was higher than that of MNA-SF (*Z* = 2.785, *P* = 0.005). The screening specificity of MNA-SF was higher than PhA and combined detection of PhA and MNA-SF for possible sarcopenia and the difference was statistically significant (Table 5 and Fig. 1).

**Table 5:** The screening ability analysis of PhA and MNA-SF single or combined detection for possible sarcopenia

Diagnostic methods	AUC (95%CI)	Cutoff value	Sensitivity (95%CI)	Specificity (95%CI)	*Z*	*P*
**Pha**	0.640 (0.573,0.702)	4.5	58.49 (48.5,68.0)	66.67 (57.5,75.0)	2.804^a^	0.005
**MNA-SF**	0.505 (0.438,0.572)	9	94.0 (2.0–51)	100.00 (97.0–100.0)	2.785^b^	0.005
**Combined detection**	0.642 (0.575,0.704)	0.503	57.55 (47.6,67.1)	70.00 (61.0,78.0)	1.101^c^	0.271

^a^The comparison of ROC area between PhA and MNA-SF; ^b^The comparison of ROC area between MNA-SF and combined detection; ^c^ The comparison of ROC area between PhA and combined detection

### The screening ability analysis of PhA and MNA-SF in screening of pre-sarcopenia

**Fig. 2:**
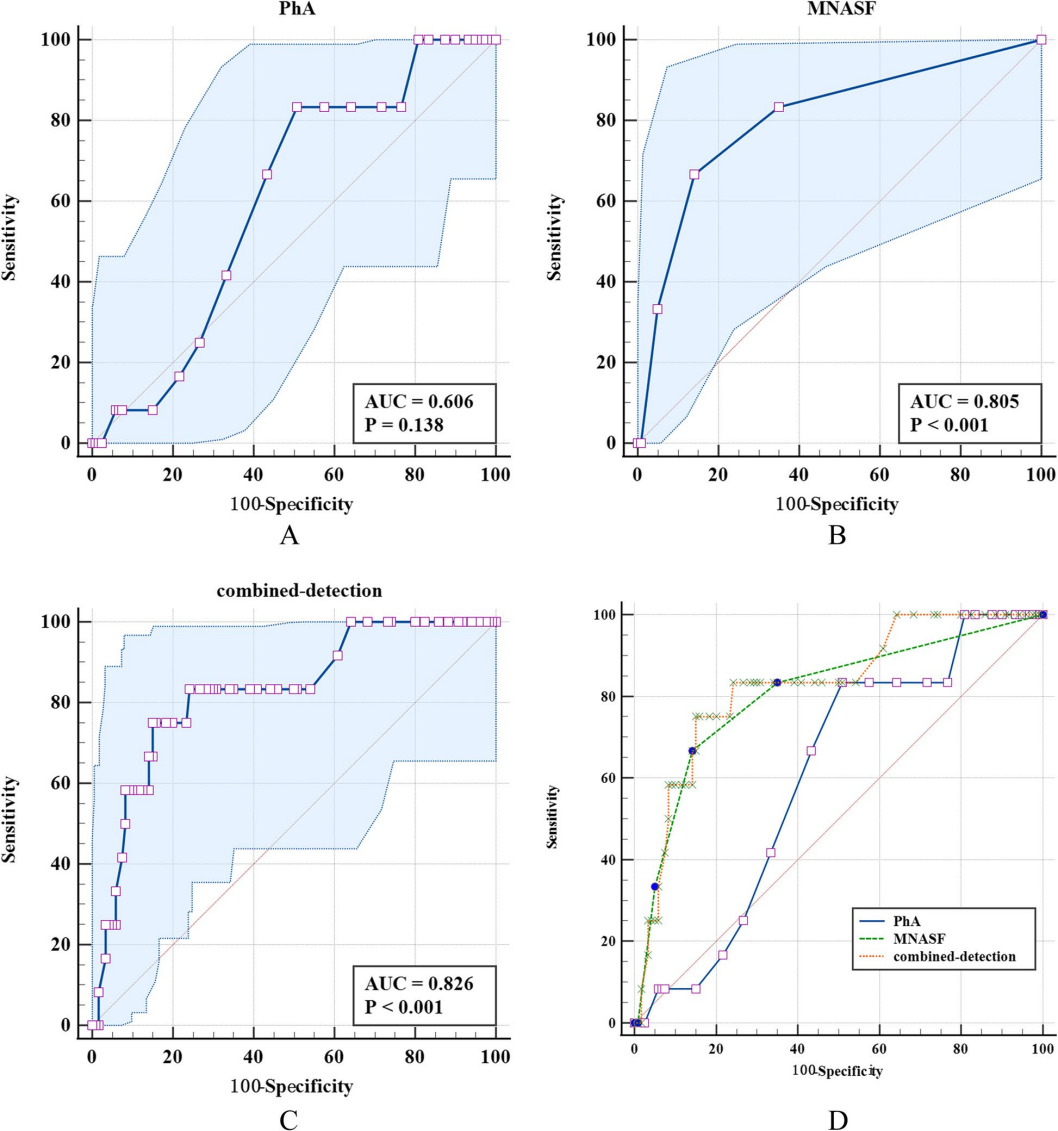
The area under the ROC curve of PhA and MNA-SF single or combined detection for the screening of pre-sarcopenia. Note: **A** is the ROC curve for the screening of pre-sarcopenia by PhA, **B** is the ROC curve for the screening of pre-sarcopenia by MNA-SF, **C** is the ROC curve for combined detection for screening of pre-sarcopenia，**D** is the ROC curve for the screening of pre-sarcopenia by PhA, MNA-SF and the combined detection

Compared with PhA, MNA-SF was valuable in the screening of pre-sarcopenia (AUC was 0.805, *P* < 0.05). The ROC area of combined detection of PhA and MNA-SF was higher than that of the single detection of PhA in pre-sarcopenia (*Z* = 2.454, *P* = 0.014). The screening specificity of MNA-SF in the detection of pre-sarcopenia was higher than that of the PhA, the difference was statistically significant. The screening specificity of combined detection of PhA and MNA-SF for pre-sarcopenia was higher than that of the single detection of PhA, the difference was statistically significant (Table 6 and Fig. 2).

**Table 6:** The screening ability analysis of PhA and MNA-SF single or combined detection for pre-sarcopenia

Diagnostic methods	AUC (95%CI)	Cutoff value	Sensitivity (95%CI)	Specificity (95%CI)	*Z*	*P*
**PhA**	0.606 (0.517,0.689)	4.7	83.33 (51.6,97.9)	49.17 (39.9,58.4)	1.934^a^	0.053
**MNA-SF**	0.805 (0.727,0.869)	12	66.67 (34.9,90.1)	85.83 (78.3,91.5)	1.360^b^	0.174
**Combined detection**	0.826 (0.750,0.886)	0.128	75.00 (42.8,94.5)	85.00 (77.3,90.9)	2.454^c^	0.014

^a^The comparison of ROC area between PhA and MNA-SF; ^b^The comparison of ROC area between MNA-SF and combined detection; ^c^The comparison of ROC area between PhA and combined detection

### The screening ability analysis of PhA and MNA-SF in screening of sarcopenia (common type)

**Fig. 3:**
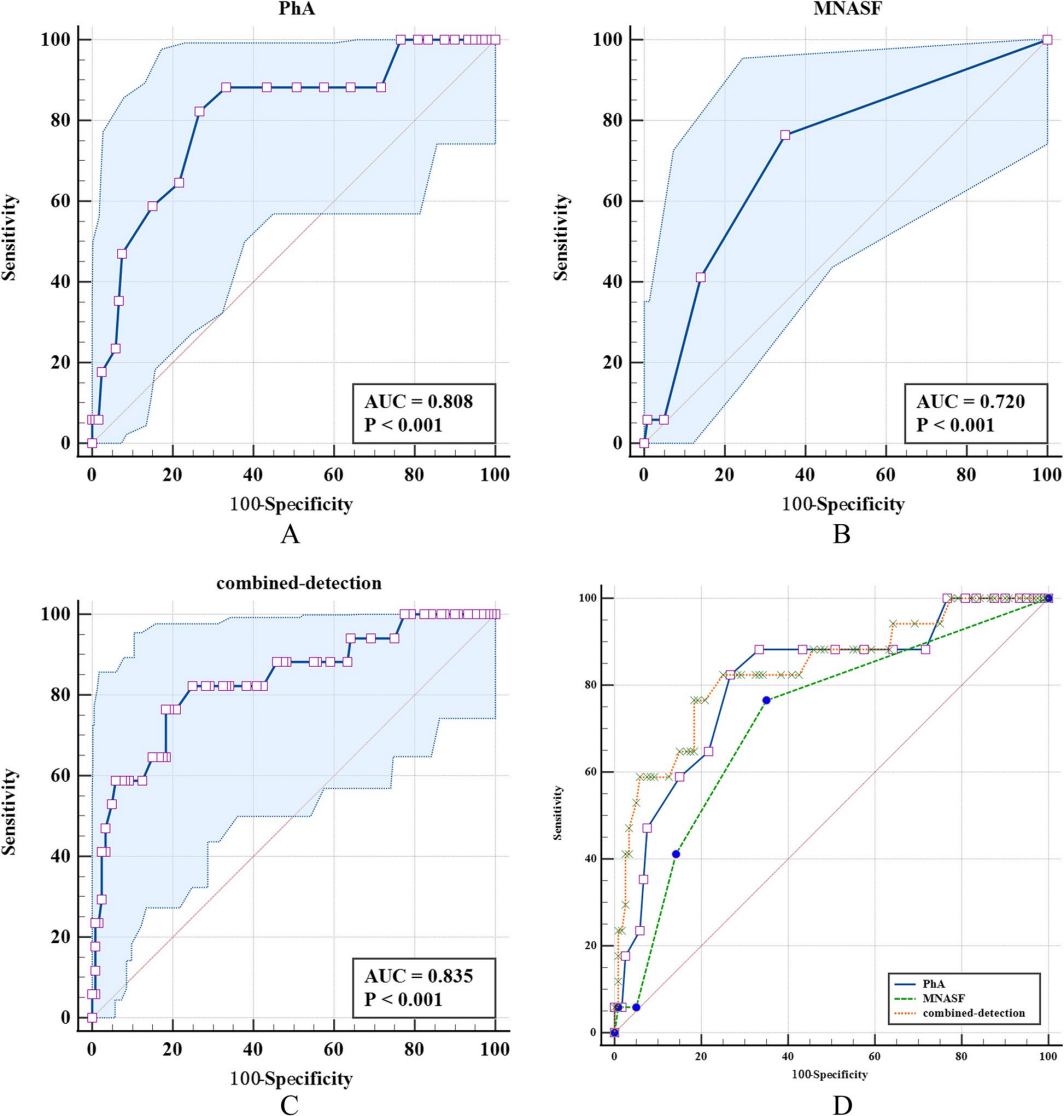
The area under the ROC curve of PhA and MNA-SF single or combined detection for the screening of sarcopenia (common type). Note: **A** is the ROC curve for the screening of sarcopenia (common type) by PhA, **B** is the ROC curve for the screening of sarcopenia (common type) by MNA-SF, **C** is the ROC curve for combined detection for diagnosis of sarcopenia (common type)，**D** is the ROC curve for the diagnostic of sarcopenia (common type) by PhA, MNA-SF and the combined detection

The PhA and MNA-SF all had the screening ability in the sarcopenia (common type) and the area under the ROC curve was 0.808 and 0.720. The ROC area of combined detection was higher than the MNA-SF (*Z* = 2.055*, P* = 0.039). The screening specificity of combined detection of PhA and MNA-SF for sarcopenia (common type) was higher than that of the single detection of MNA-SF (Table 7 and Fig. 3).

**Table 7:** The screening ability analysis of PhA and MNA-SF single or combined detection for sarcopenia (common type)

Diagnostic methods	AUC (95%CI)	Cutoff value	Sensitivity (95%CI)	Specificity (95%CI)	*Z*	*P*
**Pha**	0.808 (0.732,0.870)	4.4	82.35 (56.6,96.2)	73.33 (64.5,81.0)	1.200^a^	0.230
**MNA-SF**	0.720 (0.637,0.794)	13	76.47 (50.1,93.2)	65.00 (55.8,73.5)	2.055^b^	0.039
**Combined detection**	0.835 (0.762,0.893)	0.151	76.47 (50.1,93.2)	81.67 (73.6,88.1)	1.125^c^	0.260

^a^The comparison of ROC area between PhA and MNA-SF; ^b^The comparison of ROC area between MNA-SF and combined detection; ^c^The comparison of ROC area between PhA and combined detection

### The screening ability analysis of PhA and MNA-SF in screening of severe sarcopenia

**Fig. 4:**
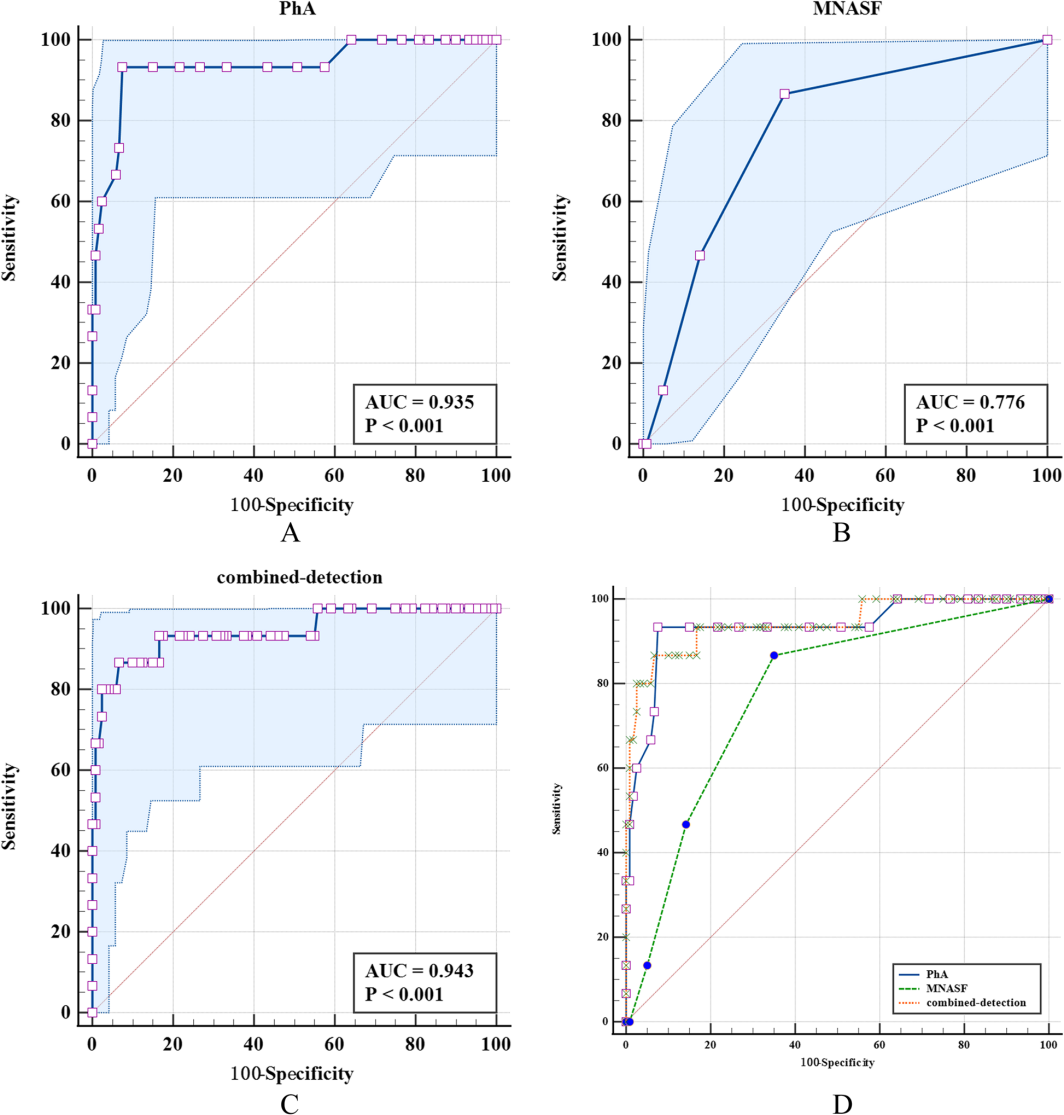
The area under the ROC curve of PhA and MNA-SF single or combined detection for the screening of severe sarcopenia. Note: **A** is the ROC curve for the screening of severe sarcopenia by PhA, **B** is the ROC curve for the screening of severe sarcopenia by MNA-SF, **C** is the ROC curve for combined detection for diagnosis of severe sarcopenia，**D** is the ROC curve for the diagnostic of severe sarcopenia by PhA, MNA-SF and the combined detection

The area under the ROC curve of PhA was higher than of MNA-SF (*Z* = 2.318, *P* = 0.020). The ROC area of combined detection was higher than the MNA-SF(*Z* = 2.730, *P* = 0.006). The screening specificity of combined detection of PhA and MNA-SF for severe sarcopenia was higher than that of the single detection of MNA-SF, and the screening specificity of PhA was higher than that of MNA-SF (Table 8 and Fig. 4).

**Table 8:** The screening ability analysis of PhA and MNA-SF single or combined detection for severe sarcopenia

Diagnostic methods	AUC (95%CI)	Cutoff value	Sensitivity (95%CI)	Specificity (95%CI)	*Z*	*P*
**Pha**	0.935 (0.879,0.970)	4.1	93.33 (68.1,99.8)	92.50 (86.2,96.5)	2.318^a^	0.020
**MNA-SF**	0.776 (0.696,0.843)	13	86.67 (59.5,98.3)	65.00 (55.8,73.5)	2.730^b^	0.006
**Combined detection**	0.943 (0.890,0.976)	0.170	86.67 (59.5,98.3)	93.33 (87.3,97.1)	0.722^c^	0.470

^a^The comparison of ROC area between PhA and MNA-SF; ^b^The comparison of ROC area between MNA-SF and combined detection, ^c^The comparison of ROC area between PhA and combined detection

## Discussion

Sarcopenia is a common disease among the older adults. As the world’s population is ageing, there will be higher prevalence of sarcopenia in older adults. In this study, it was discovered that the severity of sarcopenia aggravates as people get older. The PhA and MNA-SF scores were different in different stages of sarcopenia. PhA and MNA-SF were positively correlated with skeletal muscle mass index, grip strength, walking speed, SPPB scores, and body function. Both PhA and MNA-SF could effectively identify sarcopenia among the older adults in the community. The screening accuracy of PhA was higher than that of MNA-SF. At present, there is no study comparing the value of MNA-SF and PhA in identifying different sarcopenia stages, such as pre-sarcopenia and possible sarcopenia. By analyzing different stages of sarcopenia, it was found that the PhA has the value of screening possible sarcopenia and sarcopenia (common type), while MNA-SF has a great screening value in pre-sarcopenia. The PhA and MNA-SF both played a role in the screening of severe sarcopenia. This provided reference for the timely screening of patients with sarcopenia in different stages in the community.

The PhA is directly related to the volume of intracellular fluid and is closely related to muscle tissue, which may explain that individuals with higher muscle mass may also have higher PhA [26]. Phase angle is also significantly correlated with grip strength and other motion indexes. High phase angle is significantly positively correlated with grip strength, knee extension force, and high activity level of masters [27]. Similar to the results obtained in this study, the reason behind is mainly muscle mass and decrease in muscle strength of people with low phase angle, which further leads to the decrease of body function performance [28]. Although the possible sarcopenia patients have not yet developed to the sarcopenia, the reduce grip strength and body function can also cause decreased exercise ability among the older adults, affecting the quality of life. This study showed that the PhA had certain significance in the screening of possible sarcopenia, but the screening ability was not high, which was mainly due to the fact that the possible sarcopenia patients only experience a decrease in grip strength and body function without a loss of skeletal muscle mass. It indicated that the nutritional status of the possible sarcopenia has not yet changed significantly, and the decline function may be related to fewer physical activities. Health education for the patients with possible sarcopenia should be prioritized in the community work, so as to prevent them from developing sarcopenia. The diagnostic criteria of possible sarcopenia according to AWGS2019, included grip strength or/and walking speed. While grip strength was only one of the indicators reflecting muscle strength, it was reported that back muscle strength was also an important indicator of muscle strength [29]. In the present study, only grip strength was detected, which does not fully reflect the muscle strength level of the older adults. It was necessary to comprehensively consider various factors and indicators that affect muscle strength in the future study.

Mainly charactered by decreased muscle mass, pre-sarcopenia may be related to insufficient protein synthesis caused by the poor nutritional status of patients. As the largest protein pool in human body, skeletal muscle accounts for about 60% of the total protein and 20% of the protein weight of muscle. With the increasing age, protein synthesis reaction decreases, resulting in the decline of muscle mass [30]. As a tool for screening nutritional status of the older adults, MNA-SF delivered the same results of malnutrition with that obtained according to the laboratory standards. Therefore, malnutrition in patients with chronic diseases can be better identified [31]. In addition, MNA-SF also has good application value due to its role in reducing the rate of missed diagnosis while screening protein energy consumption [32]. This study also found that MNA-SF scores was mainly positively correlated with SMI, and that MNA-SF had a high value in the screening of pre-sarcopenia. This finding could provide a certain reference for the community to carry out large-scale screening of pre-sarcopenia. In the screening of pre-sarcopenia, it was found that the screening ability of PhA combined with MNA-SF was higher than that of PhA alone, with a sensitivity of 75.00%, indicating that the combination of PhA and MNA-SF could identify 75.00% of the patients with pre-sarcopenia, while 25.00% of them might be negative (a false negative rate of 25.00%). Furthermore, PhA combined with MNA-SF could also identify 85.00% (specificity) of the patients without pre-sarcopenia and 15.00% (a false positive rate) of older adults were classified as pre-sarcopenic patients. This study aimed to identify patients with pre-sarcopenia as early as possible to take measures. Therefore, PhA combined with MNA-SF was preferred in screening the patients with pre-sarcopenia.

The PhA and MNA-SF indicated that nutritional status was closely related to sarcopenia components. In this study, it was found that the PhA was positively correlated with SMI, grip strength, 6 m gait speed, and SPPB scores, indicating that the change of phase angle could lead to changes in skeletal muscle mass, strength, and function. The PhA reflects the quantity and quality of soft tissue, nutritional status, and body function. When the nutrient intake is insufficient or protein deficiency is caused, the phase angle will be reduced. Our results also indicated that the PhA had a good application value in the screening of sarcopenia in the community, which is consistent with the results of Chen Xinyu et al. [13]. The MNA-SF scale is widely used in the survey of nutritional status of patients with various chronic diseases because it is inexpensive and easy to use. Tan VMH et al. [33] found that MNA-SF can identify micronutrient deficiency caused by unbalanced diet, which further lead to decreased muscle mass and strength [34]. It is also found in this research that the lower MNA-SF scores indicated malnutrition, which leads to the decline in muscle mass and strength and gradually develops into sarcopenia. Therefore, the PhA and MNA-SF both played a role in the screening of older adults with sarcopenia in the community.

Without timely treatment for the sarcopenia (common type), patients will suffer severe sarcopenia, which is mainly manifested by the simultaneous reduction in muscle mass, muscle strength and body function. Our study also found that patients with severe sarcopenia had lower PhA and MNA-SF scores than those in other stages, indicating a continuous decline in nutritional status. Both PhA and MNA-SF can effectively identify severe sarcopenia. Therefore, patients with severe sarcopenia should be should be provided with nutritional and medical treatment to alleviate the disease.

There were several limitations in our study. Firstly, the number of patients with pre-sarcopenia and sarcopenia (common type) and severe sarcopenia is small, so the sample size should be expanded for future study. Secondly, as a single-center, cross-sectional study, the results obtained only reflect the situation at the certain time and cannot determine the chronological sequence between malnutrition and sarcopenia. Thirdly, it is known that BIA is a technique for tests by introducing alternating current signals into the human body. The results of a test are susceptible to the unevenly distributed current and the different postures during the test. The subjects included in this study are older adults who cannot maintain a fixed posture for a while during the test. In addition, the results are also affected by the hydration status of the body. As a questionnaire, MNA-SF mainly asks subjects about their situation in the past month, which is highly influenced by the subjective feelings and recall bias of the older adults, and thus the results might also be affected [35]. Fourthly, Hormones, protein supplements, and vitamin D were not significantly different among the groups at baseline, but other medications which may affect muscle mass were not fully included. In addition, we found differences in age, sex, and comorbidities among the groups at baseline, which were the potential influencing factors.

In conclusion, PhA and MNA-SF were correlated with community sarcopenia and its components, and both the PhA and MNA-SF showed a certain value in the screening of sarcopenia in different stages. Moreover, the cutoff value for possible sarcopenia and pre-sarcopenia was put forward. The screening ability of PhA alone or in combination was higher than that of MNA-SF in the screening of possible sarcopenia. The combination of PhA and MNA-SF performed better than the PhA or MNA-SF alone in the screening of pre-sarcopenia. The combination of PhA and MNA-SF or PhA alone all performed better value in the screening of sarcopenia (common type). Compared to MNA-SF, the PhA performed better in the screening of severe sarcopenia. BIA and MNA-SF are two indicators adopted in this paper to identify patients with sarcopenia in different stages. As a quantitative evaluation method, BIA was cheap and portable, which was more easily accepted by the older adults. MNA-SF was a questionnaire with six questions, which required less time to conduct surveys among the older adults. Both of the two methods were highly practical in the community. The test of the PhA and MNA-SF can help community workers identify the stage of sarcopenia and provide scientific prevention and control measures, which have important public health significance. As the results mentioned above are obtained from our research on the older adults in the community of China, the relevant researches targeting different groups in other countries or regions are needed to verify the results.

## Data Availability

Data will be available upon request from the corresponding author.
